# Evaluation of the Efficacy of Antioxidant Extract from Lemon By-Products on Preservation of Quality Attributes of Minimally Processed Radish (*Raphanus sativus* L.)

**DOI:** 10.3390/antiox12020235

**Published:** 2023-01-20

**Authors:** Angela Zappia, Angelica Spanti, Rossella Princi, Valeria Imeneo, Amalia Piscopo

**Affiliations:** 1 Department of AGRARIA, University Mediterranea of Reggio Calabria, Vito, 89124 Reggio Calabria, Italy; 2 Department of Food, Environmental and Nutritional Sciences (DeFENS), University of Milan, Via G. Celoria 2, 20133 Milan, Italy

**Keywords:** citrus byproduct, coating, dipping, minimally processed vegetables, radish, phenolic compounds

## Abstract

The aim of this work was to enhance the use of a food-grade antioxidant extract obtained from lemon processing byproducts (peel, pulp and seeds) to extend the shelf life of minimally processed radishes. The extract (LP_E_) was previously characterized in terms of total phenolic (6.75 ± 0.34 mg GAE g^−1^ d.w.) and flavonoid content (2.04 ± 0.09 mg CE g^−1^ d.w.) and antioxidant activity, and eriocitrin and hesperidin were identified as the most prevalent phenolic compounds by a UHPLC system. The effects of different dipping aqueous solutions (UCR, DRa, DRb) and alginate-based edible coating formulations (CRc, CRd) with and without the antioxidant extract were studied on the quality parameters of minimally processed radishes, characterized regarding their microbiological and physicochemical characteristics for up to 14 days at 3 °C. The coating formulated with LP_E_ delayed the radish respiration process, as well as resulting in less color variation (ΔE < 3) and reduced mesophilic aerobic count values (4.49 ± 1.43 log CFU g^−1^), proving the effectiveness of LP_E_ as a value-added ingredient in developing post-harvest strategies to prolong the shelf life of minimally processed vegetables. Indeed, coated samples without the extract showed a clear development of rotting, which led to the end of their shelf life on their 7th day of storage.

## 1. Introduction

Minimally processed vegetables provide consumers with a high content of bioactive phenolic compounds and nutritional properties. The consumption of vegetables is widespread because it can reduce the incidence of many diseases, such as cancer and cardio- and cerebrovascular diseases, thanks to their high content of antioxidant compounds [1,2].

In this context, radish (*Raphanus sativus L.*) is a vegetable recognized worldwide for its many bioactive compounds such as anthocyanins, flavonoids, phenols, vitamins and pigments, which affect its appearance and nutritional quality. Indeed, radish leaves and roots have been therefore used in various parts of the world for medical applications due to their antimicrobial, antiviral and antioxidant activity [3,4]. The dietary consumption of fresh radish is also an excellent source of bioactive and useful compounds for human health. In this regard, it is well known that minimal processing operations, such as cutting or peeling, could damage the integrity of vegetable tissues, triggering deterioration processes including oxidative browning, tissue softening, water loss and the production of undesirable flavors and odors [5]. The greatest losses in the quality and quantity of fresh fruits and vegetables occur from harvest to consumption, due to the change in the gas balance between oxygen consumption and carbon dioxide production by the plants [6]. The gas transfer rate depends on internal and external factors, such as cultivar or atmospheric composition, in terms of the O_2_, CO_2_ and ethylene ratio [7]. In this context, the main objectives of any post-harvest technology are quality optimization and loss reduction in fresh produce [8]. Controlled and modified atmosphere packaging or dipping with natural additives, such as salts or organic acids (e.g., calcium chloride or citric acid and ascorbic acid), have been used for preserving different minimally processed vegetables and for reducing variations in the quality and quantity of their components [9,10]. In addition, edible coatings represent a new packaging strategy in the post-harvest management of fresh produce, as primary packaging on the surface of vegetables or fruits. They can provide an alternative to modified atmosphere packaging, maintaining the original quality by modifying and controlling the internal atmosphere of the individual fruit or vegetable. Edible coatings can reduce moisture and solute migration, gas exchange, respiration and oxidative reaction rates and could be considered as potential carriers of active ingredients such as antibrowning agents, colorants, flavorings, nutrients and antimicrobial compounds, which can extend product shelf life and reduce the risk of microbiological growth [6,11]. Edible coating formulations are based on materials with film-forming ability, and plasticizers, antimicrobial agents, minerals, vitamins, colors or flavorings may be included [12]. In the last decade, interest has grown in the use of edible coatings on fruits and vegetables to extend their post-harvest quality and shelf life [13,14,15,16].

Moreover, the use of food byproduct extracts could be a valid way to incorporate active compounds with significant antioxidant and antimicrobial activity in edible coating formulations [17]. In this view, citrus processing industries produce huge amounts of waste and byproducts every year [18]. High value-added molecules can be extracted from citrus byproducts and applied in various commercial sectors, such as food, cosmetics and pharmaceuticals [19,20]. The main bioactive compounds found in citrus processing byproducts are essential oils, flavonoids, carotenoids, limonoids, phenolics, organic acids, vitamins, pectins and enzymes, which show a great potential to be used as natural antioxidants and antimicrobials in food production and as active agents in dipping solutions or edible coating formulations with the aim to preserve and extend the shelf life of minimally processed fruits and vegetables [21,22,23]. Specifically, lemon byproducts (peel, pulp and seeds) are considered a significant source of bioactive compounds, among which flavanones and flavones are the most abundant flavonoids, followed by neohesperidin, naringin, rutin and apigenin. An equally considerable amount of phenolic acids was also detected, such as gallic acid, protocatechic acid, p-cumaric acid and obacunone [24,25,26,27,28,29].

In this view, the aim of this work was to investigate the efficacy of a dipping solution and an alginate-based coating supplemented with a lemon byproduct phenolic extract on minimally processed radishes, to assess the quality preservation and shelf-life extension of the final product in refrigerated storage conditions, while enhancing the potential of a citrus industry byproduct. The use of these food wastes not only reduces their considerable environmental impact, but also promotes a circular economy by favoring the recovery of highly available, low-cost and natural substances beneficial to human health in which they are plentiful. Furthermore, the formulation of edible coatings with food-grade ingredients provides a concrete response to the growing consumer demand for safe and ready-to-eat products with an appropriate shelf life, with a further food waste reduction.

## 2. Materials and Methods

### 2.1. Raw Material

Lemon byproduct samples (*Citrus limon (L.) Osbeck*) containing peels, pulp and seeds were supplied by the Agrumaria Reggina company located in Gallico (Reggio Calabria, Italy) after the extraction of lemon juice and essential oils. Lemon byproducts were transported to the Food Technology Laboratory of the University Mediterranea of Reggio Calabria, immediately dried at a temperature of 50 °C up to a final moisture content of 12% and stored in polyethylene bags under a vacuum to avoid rehydration until the subsequent extraction procedures of the bioactive compounds.

Radishes were supplied by a local distributor in the province of Reggio Calabria (Italy) and transported to the Food Technology Laboratory of the University Mediterranea of Reggio Calabria, where leaves were completely removed before subjecting the radish roots to processing.

### 2.2. Preparation of Lemon Byproduct Phenolic Extract (LP_E_)

The lemon byproduct extract (LP_E_) used in this study was obtained by an ultrasound-assisted extraction at 25 °C for 60 min using a hydroalcoholic mixture (50%) as the extraction solvent, as reported and characterized by Imeneo et al. [30].

### 2.3. Preparation of Radish Sample

**Figure 1:**
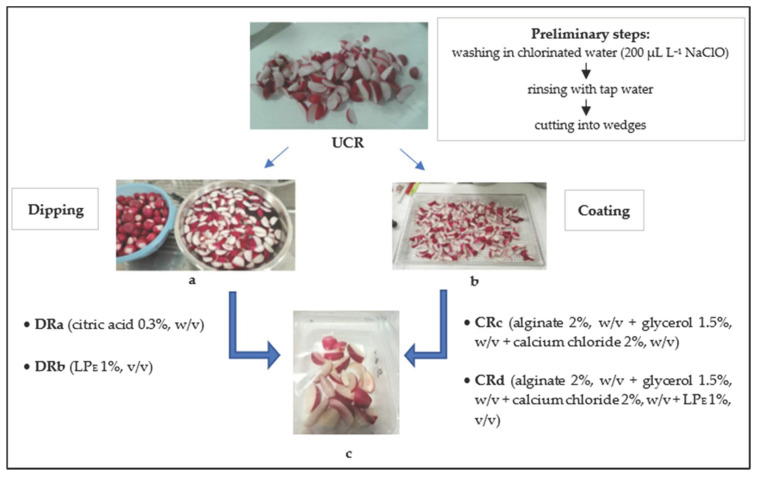
Minimal processing steps for cut radish treatments: (**a**) dipping, (**b**) coating and (**c**) packaging. Abbreviations: UCR, uncoated minimally processed radishes; DRa, minimally processed radishes dipped in 0.3% citric acid solution, *w*/*v*; DRb, minimally processed radishes dipped in 1% LP_E_ solution, *v*/*v*; CRc, minimally processed radishes coated with an alginate-based coating (2%, *w*/*v*); CRd, minimally processed radishes coated with an alginate-based coating (2%, *w*/*v*) with LP_E_ (1%, *v*/*v*).

Radish roots were subjected to a first washing in chlorinated water (200 µL L^−1^ NaClO) for two minutes and then rinsed with tap water. After the washing and removal of excess water at room temperature, the radishes were cut into wedges to obtain a UCR sample (uncoated radishes). Successively, an aliquot of minimally processed radishes was submitted to dipping and coating treatments, according to Oms-Oliu et al. [1]. For the dipping treatments, a portion of minimally processed radishes was dipped in a 0.3% (*w*/*v*) citric acid solution or an aqueous solution containing the LP_E_ (1%, *v*/*v*) to produce the samples DRa and DRb, respectively. Coating solutions were prepared by dissolving alginate (Sigma-Aldrich Chemic, Steinhein, Germany) in distilled water (2%, *w*/*v*) at 70 °C under continuous stirring until the solution became clear. Glycerol (Carlo Erba Reagents S.r.l., Italy) was added as a plasticizer (1.5%, *w*/*v*) in the alginate solution and the radish pieces were dipped in it for 2 min. The excess coating material was drained for 1 min. For the crosslinking alginate, a calcium chloride solution (2%, *w*/*v*) was prepared and the radish pieces were dipped into it for 2 min to collect the sample CRc. Similarly, another calcium chloride solution (2%, *w*/*v*) containing the LP_E_ (1%, *v*/*v*) was prepared for the formulation of the CRd sample. All the treated and minimally processed radishes (60 g) were packaged in polypropylene trays and closed with a polypropylene/polyethylene terephthalate film on top using a thermosealed machine (VGP 25n, ORVED) and stored at 3 °C for 14 days (Figure 1). At every monitoring time, two trays of each treatment were picked up to perform the repetition of analyses.

### 2.4. Headspace Gas Composition

The respiratory rate was determined according to the method reported by Del Aguila et al. [31]: 15 g of minimally processed radishes was placed in sealed glass jars of 300 mL equipped with a rubber septum to insert the needle for the measurement of the CO_2_ and O_2_ percentages using a CheckPoint handheld gas analyzer (PBI Dansensor, Milan, Italy). The first measurement (time zero) was carried out 1 h after processing and after 1, 3 and 7 days. Results were expressed in CO_2_% and O_2_%.

### 2.5. Microbiological Analysis

For the microbiological analyses, the total aerobic count (TBC) was determined. An aliquot of minimally processed radishes (10 g) was diluted with a sterile Ringer’s solution and homogenized with a Stomacher (BagMixer^®^; Interscience, Saint-Nom-la-Bretèche, France) for 2 min. Serial decimal dilutions were prepared and plated on Petri plates with a plate count agar (PCA) growth medium (Oxoid, Milan, Italy) for TBC at 26 °C for 48 h. The results were expressed as the log CFU g^−1^ [32].

### 2.6. Physicochemical Analysis

The samples were submitted to the following determinations: titratable acidity (% of citric acid) according to the AOAC method [33]; pH by pH meter (Crison GLP, Barcelona, Spain); total soluble solids (°Brix) using a digital refractometer (PR-201a; Atago); and dry matter (%) as weight loss in an oven at 70 °C until a constant weight was reached, according to the AOAC method [34].

Color analyses were performed with a tristimulus colorimeter (model CM-700d; Konica Minolta, Osaka, Japan) on the outer and inner portions of the minimally processed radishes and were referred to the CIELAB color space for the parameters L*, a* and b*. Color changes in the minimally processed radishes were measured by the hue angle (h°) parameter, according to Oms-Oliu et al. [35]:

h° = arctan b*/a*(1)

The total color difference (ΔE), in the outer and inner sides of the minimally processed radishes on the 1st day and after 14 days of storage, was obtained by the following formula, according to Thompson [36]:

(2) $$\Delta E=\sqrt{\left[{\left(L-L_{0}\right)}^{2}+{\left(a-a_{0}\right)}^{2}+{\left(b-b_{0}\right)}^{2}\right]}$$

where L*_0_, a*_0_ and b*_0_ are the initial considered values (1st day).

### 2.7. Total Polyphenol and Anthocyanin Determinations

The minimally processed radishes were submitted to methanolic extraction, according to Marotti and Piccaglia [37]. In brief, 10 g of the sample was homogenized with 25 mL of methanol:water:acetic acid (50:42:8, v:v:v) for 2 min, centrifuged at 5000× *g* for 10 min at 4 °C. The supernatant was collected and residues were re-extracted. Both supernatant solutions were filtered through syringe filters (0.45 µm Chromafil RC-45/25), combined and diluted up to 25 mL in volume with extraction solution.

Total phenolic content (TPC) was determined according to the method by Singleton and Rossi [38] using Folin–Ciocalteu reagent (Carlo Erba, Milan, Italy), by reacting 100 µL of the previously obtained methanolic extract. The solutions were spectrophotometrically analyzed at 760 nm in a UV–VIS spectrophotometer (Agilent, Santa Clara, California, USA) and the results were reported as mg of gallic acid kg^−1^ of radish fresh weight (mg GAE kg^−1^).

The total anthocyanin content (TAC) was determined spectrophotometrically according to the AOAC method [39] with the methanol extract diluted (D = 1:5, v:v) with a pH 1.0 buffer (potassium chloride, 0.025 M) and pH 4.5 buffer (sodium acetate, 0.4M), and the absorbance was determined against the blank (distilled water) at both 520 and 700 nm. Anthocyanin pigment concentration was expressed as mg of cyaniding 3-glucoside kg^−1^ of radish fresh weight (mg C-3-GLUC kg^−1^) and calculated as follows:

(3) $$\frac{A\times MW\times DF\times 10^{3}}{\varepsilon \times 1}$$

where A = (A520 nm–A700 nm) pH 1.0–(A520 nm–A700 nm) pH 4.5; molecular weight (MW) = 449.2 g mol^−1^ for cyaniding 3-glucoside (cyd-3-glu); DF = dilution factor established in D; 1 = pathlength in cm; ε = 26900 molar extinction coefficients, in L mol^−1^ cm^−1^, for cyd-3-glu; and 10^3^ = factor for conversion from g to mg.

### 2.8. Antioxidant Activity

For determining the antioxidant activity of the minimally processed radishes, DPPH and ABTS radical scavenging assays were performed. The first assay was evaluated according to the method by Re et al. [40]: 2975 µL of ABTS solution in ethanol and 25 µL of methanolic extract (Section 2.7) were mixed and the absorbance was read spectrophotometrically at 734 nm after 6 min. The DPPH radical scavenging activity was determined by using DPPH methanolic solution, according to the method by Brand-Williams et al. [41]: 2950 µL of DPPH solution and 50 µL of methanolic extract (Section 2.7) were mixed, kept in the dark for 15 min and the absorbance was recorded at 515 nm using a spectrophotometer. The results were expressed as the inactivation percentage (inactivation %).

### 2.9. Identification and Quantification of Antioxidant Compounds in Minimally Processed Radishes

The analysis was carried out for each minimally processed radish sample as reported by Romeo et al. [42], with a few modifications. The chromatographic system comprised a PLATINblue UHPLC (Knauer, Berlin, Germany) equipped with a binary pump system, a Knauer blue orchid C18 column (1.8 mm, 100 × 2 mm) combined with a PLATINblue (Knauer, Berlin, Germany) PDA-1 (photodiode array detector) and Clarity 6.2 software. 

The extracts (Section 2.7) were filtered with 0.22 µm nylon syringe filters (diameter 13 mm) and then 5 µL was injected in the system. Water acidified with acetic acid (A, pH 3.10) and acetonitrile (B) comprised the mobile phases used. The gradient elution program consisted of the following: 0–3 min, 5% B; 3–15 min, 5–40% B; and 15–15.5 min, 40–100% B. Finally, a return to initial conditions was achieved during the analysis by holding the column at 30 °C. External standards (concentration between 1 and 100 mg kg^−1^) were used for the quantification of each antioxidant compound and the results were reported as mg kg^−1^ of radish dry weight.

### 2.10. Statistical Analysis

The results were reported as the mean value ± standard deviation (mean ± SD) of three replicates. The significance of the results and statistical differences were analyzed using SPSS software (version 15.0; SPSS Inc., Chicago, IL, USA). Analyses of variance (ANOVA and multivariate analysis) were performed to compare the mean values of the different preservative treatments and Tukey’s multiple range elaboration was used as a post hoc test (*p* < 0.05). Pearson’s correlation test was employed for the determination of correlation coefficients (r) among the extracted polyphenolic and anthocyanin compounds and antioxidant assays.

## 3. Results and Discussion

### 3.1. Antioxidant Lemon Byproduct Extract Characterization

The antioxidant profile characterizations of the LP_E_ used as the functionalized dipping solutions and coating formulations are reported in Table 1.

The extract was characterized in terms of total polyphenols and flavonoid content, antioxidant activity and with regards to the identification and quantification of the main antioxidant compounds, performed by the UHPLC system. Among the various bioactive compounds found in LP_E_, eriocitrin and hesperidin were identified as the most plentiful flavonoids with a content of 33.4 ± 0.14 and 47.2 ± 0.19, respectively, followed by gallic acid, narirutin, p-cumaric acid, neoeriocitrin, naringin, rutin and ferulic acid. These compounds are known for their significant antioxidant and antimicrobial activity, with well-known beneficial effects on human health [30,43].

### 3.2. Headspace Gas Composition

**Figure 2:**
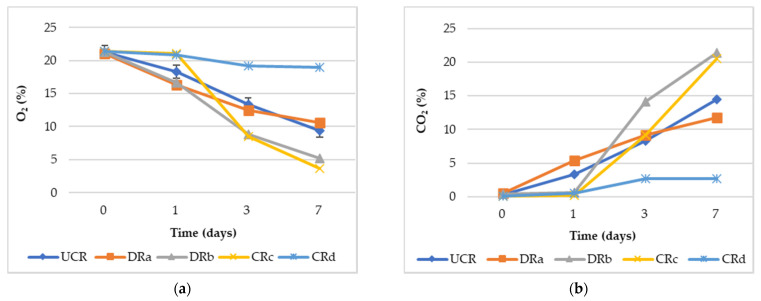
(**a**) Headspace gas composition values (O_2_%) of uncoated and coated minimally processed radishes throughout 7 days of storage at 3 °C. (**b**) Headspace gas composition values (CO_2_%) of uncoated and coated minimally processed radishes throughout 7 days of storage at 3 °C. Abbreviations: UCR, uncoated minimally processed radishes; DRa, minimally processed radishes dipped in 0.3% citric acid solution, *w*/*v*; DRb, minimally processed radishes dipped in 1% LP_E_ solution, *v/v*; CRc, minimally processed radishes coated with an alginate-based coating (2%, *w*/*v*); CRd, minimally processed radishes coated with an alginate-based coating (2%, *w*/*v*) with LP_E_ (1%, *v*/*v*).

Gas composition (O_2_% and CO_2_%) evaluated in the headspace of the sealed glass jars is shown in Figure 2. The general trend is that the O_2_ concentrations decreased from the 1st to the 7th day of monitoring, while an opposite trend was observed for the CO_2_ concentration values, confirming what was described by different authors [44,45]. The formulation of alginate-based coating with the addition of LP_E_ (CRd) significantly (*p* < 0.01) reduced the respiration process compared to the other treated and minimally processed radishes, showing the highest O_2_% and the lowest CO_2_% values throughout monitoring. These results highlighted the effectiveness of the synergistic effect of antioxidant extract and alginate-based coating (CRd) to delay the deteriorative processes and to extend the minimally processed radishes’ shelf life. This might be attributed to the preservative action exerted by the antioxidant extract (LP_E_) or to the presence of calcium ions contained in the coating formulation on the radishes, rather than the effect of the oxygen barrier properties by the coatings, as suggested by Wong et al. [46] and Lee et al. [47] in cut apples.

Indeed, both the minimally processed radishes DRb and CRc, characterized by dipping only in LP_E_ or by the presence of the alginate-based coating, respectively, showed the lowest O_2_% and the highest CO_2_% values, demonstrating the inefficiency of these two treatments in reducing the minimally processed radishes’ respiration rate.

### 3.3. Effect of Functionalized Edible Coating on Minimally Processed Radish Quality Parameters

The results of the microbiological analysis and quality parameters of the minimally processed radishes subjected to different types of treatment during monitoring throughout 14 days of storage at 3 °C are shown in Table 2. For the microbial count, there were highly significant differences among treatments after 7 days of storage (*p* < 0.01). The lowest aerobic bacterial count was found in CRc (2.58 ± 0.16 log CFU g^−1^); this was probably because the O_2_% decrement during the storage period (Figure 2) caused an anaerobic condition inside the package, with a consequently minor aerobic bacterial growth. Despite this, the development of superficial browning and softening were the first obvious and visible consequences defining the end of CRc shelf life on the 7th day. The other samples showed a microbial count increment over time: the UCR did not significantly (*p* > 0.05) differ between the dipped and coated samples, confirming what was found in [1], not exceeding 6 log CFU g^−1^. The presence of LP_E_ in the alginate-based coating formulation (CRd) did not express a great variation in this qualitative parameter, as instead demonstrated by Rojas-Graü et al. [45] and Raybaudi-Massilia et al. [48] regarding the active coating applied to cut apples. The lack of an antimicrobial effect by LP_E_ in the dipping and coating formulations could be due to the antioxidant extract’s pool of secondary metabolites, such as tannins, flavonoids and phenolic compounds, which are known to have antimicrobial properties [43]. Indeed, as stated by Ivasenko et al. [49], the extraction method of valuable compounds from plant matrices is crucial to the composition of the final extract. In this study, the absence of phenolic compounds, such as apigenin, may have influenced the absence of antimicrobial activity by the extract. As reported by Budiati et al. [50], apigenin is characterized by significant antimicrobial activity, thanks to its ability to inhibit microbial adhesion, as well as the activity of enzymes and cellular transport proteins.

Among the quality parameters of fruits and vegetables, total soluble solids and total acidity play a crucial role as they affect taste and sensory characteristics [51]. In this study, according to the literature [52], total soluble solids increase during storage while total acidity decreases. Specifically, a highly significant reduction (*p* < 0.01) in total acidity values was noted for all samples over time, except for CRc, which showed a similar value until the 7th day of storage. An opposite trend to the one described above, except for UCR and CRd, was found for total soluble solid values, showing a significant increase over time, which might be due to the reduction in the metabolic activity of vegetables [53]. The pH significantly increased (*p* < 0.01) throughout 14 days for both coated and uncoated samples, although not showing a clear trend. The different developments of acidity and total soluble solid values could be explained by the progressive vegetable maturation process characterized by a change in the content of organic acids, which are used as respiration substrates or transformed into other compounds, resulting in taste alterations. This underlines the efficacy of LP_E_ and alginate-based coating in delaying the ripening process by lowering acidity loss because of reduced respiratory metabolism during the days of monitoring [52,54]. In addition to organic acids, sugars are an important respiration substrate, which are transformed into simpler molecules such as CO_2_ and water [55]. Indeed, the higher total soluble solid values found for CRc on the 7th day of storage could explain the sharp reduction in O_2_% and the increase in CO_2_%, as shown in Figure 2. The fact that no significant differences in total soluble solids were observed in the minimally processed radishes treated with functionalized alginate-based coating (CRd) until the last day of storage, suggests a synergistic effect of the coating and LP_E_ in reducing the carbohydrate degradation rate, thus delaying vegetable ripening [56]. 

**Figure 3:**
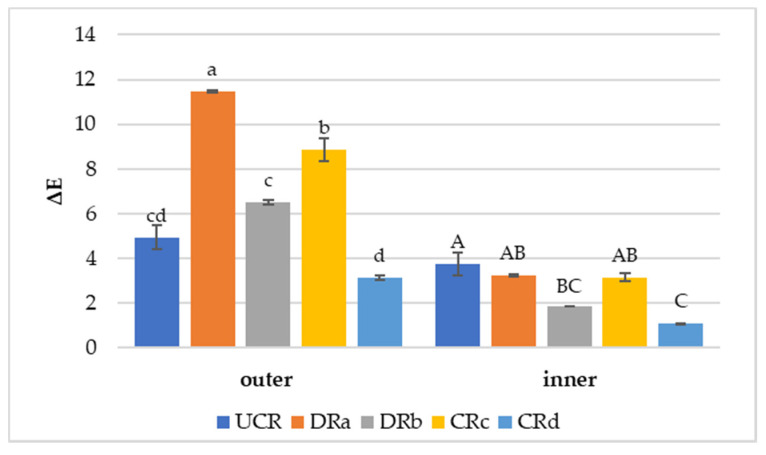
Color variation values (ΔE) of uncoated and coated minimally processed radishes between the last day (14th day) and the 1st day of storage at 3 °C. Lowercase letters indicate differences among treatments for the outer side. Uppercase letters indicate differences among treatments for the inner side. Abbreviations: UCR; DRa; DRb; CRc; CRd (see Figure 2).

When applying a coating to a fresh cut product, it is desirable that a minimum color variation occurs, as this is an attribute with a strong impact on the consumer’s perception of product quality. In this study, the color results, expressed as the hue angle (h°) and color variation (ΔE) of both the outer and inner surfaces of minimally processed radishes are reported in Table 3 and Figure 3, respectively. On the 7th day of storage, a clear increase in h° values (yellowish tonality) was observed in the inner surface of the minimally processed radishes, except for CRc. In the latter case, low h° values and a decrease over time could be attributed to the development of a darker coloration on the inner surface of the radishes due to the high degree of senescence that occurred on the 7th day of storage. Regarding the outer surface, all samples showed lower values of the h° angle than the inner side, thanks to the natural darker color of the radish’s outer surface due to the presence of acylated anthocyanins. The decreasing trend in the h° angle values is in line with the increase in pH recorded for all the samples during storage (Table 2), confirming a typical effect of acylation on the color tone on the outside of radishes [57]. In general, a gradual but non-linear reduction in values over time was observed, although the samples characterized by the presence of the extract (DRb and CRd) showed a smaller reduction than the other treatments throughout 14 days of storage.

Color variation (ΔE) results in the minimally processed radishes regarding the differences between the last day (14th day) and the 1st day of monitoring are reported in Figure 3. The use of alginate-based coating with the addition of LP_E_ (CRd) promoted a protection against color variation both on the outer and inner portions of the vegetable wedges. In fact, CRd was characterized by the lowest ΔE values of 3.13 ± 0.01 (outer) and 1.09 ± 0.01 (inner). DRb, dipped in LP_E_ solution, also exhibited a limited color variation, especially on the inner surface, suggesting that LP_E_ did not affect the natural color of the minimally processed radishes. Conversely, CRc and DRa presented the most evident color variations over time on both vegetable surfaces. Based on the color results, treatments characterized by the presence of LP_E_ (DRb and CRd) were effective in avoiding browning, confirming what was noted by Rojas-Graü et al. [58] on coated fresh cut pears. The effect shown by the alginate-based coating with LP_E_ on the radish’s color preservation indicates that the antioxidant activity of the extract could have performed more efficiently in slowing down the vegetable’s metabolic activity, such as enzymatic browning responsible for color changes, than the other coated and uncoated treatments [56].

### 3.4. Characterization of Bioactive Compounds and Antioxidant Activity of Minimally Processed Radishes

Bioactive compound (total polyphenols and anthocyanin content) and antioxidant activity results are reported in Table 4. The total polyphenol content (TPC) did not vary either among the samples or over time, contrary to what was found for the total anthocyanin content (TAC). The decrease in TAC was highly significant at the end of monitoring in CRd, with 189 ± 9 mg C-3-Glu kg^−1^. In general, looking at the data for TPC and TAC, it can be stated that the presence of the extract in the final product, whether in the form of dipping or coating formulations, did not significantly influence the total content of bioactive compounds. At the same time, comparing the two coating formulations, the presence of LP_E_ contributed to prolonging the shelf life of the vegetable beyond seven days, slowing down the phenomenon of senescence favoured by the stress condition induced by the initial processing operations and the presence of the coating itself [59].

A similar situation to that presented above was found in terms of antioxidant activity, for both the DPPH and ABTS assays. Only the use of edible coating (CRc) did not seem to substantially contribute to the enhancement of the radish’s antioxidant capacity as declared by Oms-Oliu et al. [1], contrary to what was shown in the samples with coating formulated by the addition of an ingredient with antioxidant properties, such as lemon byproduct extract [60]. A general but not significant decrease in antioxidant activity was found in all samples for the DPPH assay after seven days of storage, in contrast to the constant trend determined for the ABTS assay. As stated by Reyes et al. [61], the antioxidant activity in fruits and vegetables is correlated with the observed value in total bioactive compound content. Indeed, in this study, positive correlation values were found after 10 days of storage between DPPH scavenging and anthocyanin content (r = 0.92) and between ABTS scavenging and both polyphenolic and anthocyanin content (r = 0.75 and 0.91, respectively).

Identification and quantification of bioactive compounds on the first and the last day of monitoring in coated and uncoated minimally processed radish samples were performed by the UHPLC system (Table 5).

In this study, all coated and uncoated minimally processed radishes are characterized by a low amount of ferulic acid and a more abundant presence of flavonoids, such as quercetin and luteolin, as also reported by Pajak et al. [62] and Li et al. [63].

On the first day of monitoring, the samples characterized by the presence of the antioxidant extract (DRb and CRd) showed a significantly higher phenolic compound content than the other treated radishes, particularly regarding the amount of luteolin, with values of 21.74 ± 0.03 and 16.15 ± 0.10 mg kg^−1^, respectively. This could be attributed to the presence of the lemon antioxidant extract and its ability to counteract the action of free radicals and the subsequent oxidation of radishes’ valuable compounds, which is an aspect that is consistent with the total color difference results (Figure 3). In addition, as stated by de Oliveira et al. [64], coatings may minimize the loss of valuable compounds, preserving coated fruits and vegetables thanks to their ability to slow down enzymatic processes and thus delay changes in physicochemical characteristics related to senescence, such as color, as well as the accumulation of free radicals due to oxidative damage [65]. 

After two weeks of storage under refrigerated conditions, a progressive decrease in phenolic compounds was observed in all treated and minimally processed radishes, except for the uncoated samples UCR and DRa, for which an increase in values was recorded. This phenomenon might be linked to the occurrence of metabolic pathways that lead to the synthesis of phenolic compounds, as part of the plant’s defense strategy [66]. Indeed, an increase in phenolic compounds is one of the most studied phenomena that occurs in response to wounding in several fresh cut fruits and vegetables [21].

## 4. Conclusions

The results of this study revealed that an alginate-based edible coating might be an economical and effective carrier system for bioactive compounds, such as those identified in lemon byproduct extract in fresh cut vegetables, in order to improve their nutritional value and quality aspects.

Indeed, alginate-based coating results in a short radish shelf life due to the absence of some antioxidant agents and higher stress conditions. A higher consumption of O_2_ was observed with a reduced growth of aerobic bacteria and the appearance of a visually unappealing color. On the other hand, the use of an alginate-based coating with the lemon byproduct extract on minimally processed radishes contributed to slowing down the respiration process, limiting color variation, reducing microbial growth and improving the vegetable’s shelf life up to 14 days of storage under refrigerated conditions. In addition, the added value of the antioxidant lemon byproduct extract added in the formulation of dipping solution was highlighted by the content of bioactive compounds and their correlations with the antioxidant activity assay values and by the observed reduction in microbial load. A further positive effect of the antioxidant extract was particularly noticed in relation to the typical post-harvest respiration of minimally processed vegetables, slowing down the process and thus the degree of senescence. This allowed a better preservation of the minimally processed radishes, characterized over time by a lesser color variation, both outside and inside, thanks to a greater containment of oxidative reactions affecting polyphenolic compounds. The latter, in fact, did not show significant variations over time as well as the expression of antioxidant activity.

The coating added with an antioxidant extract from lemon byproducts represents an attractive opportunity to preserving the microbiological, physicochemical and sensorial post-harvest quality of minimally processed radishes to ensure a longer shelf life, as well as a successful strategy to valorize a food industry processing byproduct. 

## Figures and Tables

**Table 1:** Antioxidant profile characterizations of LP_E_.

TPC (mg GAE g^−1^ d.w.)	6.75 ± 0.34
TF (mg CE g^−1^ d.w.)	2.04 ± 0.09
DPPH (µmol TE g^−1^ d.w.)	8.25 ± 0.24
ABTS (µmol TE g^−1^ d.w.)	19.42 ± 0.63
Eriocitrin (mg 100 g^−1^ d.w.)	33.4 ± 0.14
Hesperidin (mg 100 g^−1^ d.w.)	47.2 ± 0.19

GAE: gallic acid equivalent; CE: catechin equivalent; TE: Trolox equivalent; TPC: total phenolic compounds; TF: total flavonoids; DPPH and ABTS: total antioxidant activity assays; d.w.: dry weight of lemon byproducts.

**Table 2:** Microbiological results and variations in acidity, pH, total soluble solids and dry matter of uncoated and coated minimally processed radishes throughout 14 days of storage at 3°C.

	Time (Days)	Samples					
		UCR	DRa	DRb	CRc	CRd	Sign.
Total aerobic bacterial count (log CFU g^−1^)	1	2.55 ± 0.55 ^C^	2.68 ± 0.11 ^C^	2.43 ± 0.20 ^B^	2.16 ± 0.35 ^B^	2.74 ± 0.26 ^B^	n.s.
	3	4.29 ± 0.56 ^B^	4.46 ± 0.66 ^AB^	4.58 ± 0.38 ^A^	3.79 ± 0.27 ^A^	4.48 ± 0.69 ^AB^	n.s.
	7	4.60 ± 0.03 ^B,a^	4.53 ± 0.70 ^AB,a^	4.38 ± 0.11 ^A,a^	2.58 ± 0.16 ^B,b^	4.22 ± 0.04 ^AB,a^	**
	10	5.27 ± 0.67 ^AB,a^	3.94 ± 0.65 ^BC,b^	4.75 ± 0.32 ^A,ab^	n.d.	5.09 ± 0.46 ^A,ab^	**
	14	6.05 ± 0.34 ^A^	5.67 ± 0.51 ^A^	5.01 ± 1.13 ^A^	n.d.	4.49 ± 1.43 ^AB^	n.s.
	SIGN.	**	**	**	**	*	
Total acidity	1	0.14 ± 0.00 ^A,a^	0.10 ± 0.01 ^A,c^	0.11 ± 0.01 ^A,bc^	0.13 ± 0.00 ^ab^	0.12 ± 0.02 ^A,abc^	**
	3	0.08 ± 0.02 ^B,bc^	0.07 ± 0.00 ^B,c^	0.07 ± 0.00 ^B,c^	0.13 ± 0.00 ^a^	0.10 ± 0.01 ^AB,b^	**
	7	0.07 ± 0.00 ^B,c^	0.10 ± 0.00 ^A,ab^	0.08 ± 0.02 ^B,bc^	0.12 ± 0.00 ^a^	0.09 ± 0.00 ^B,bc^	**
	10	0.06 ± 0.03 ^B^	0.08 ± 0.00 ^B^	0.08 ± 0.00 ^B^	n.d.	0.09 ± 0.00 ^B^	n.s.
	14	0.08 ± 0.02 ^B,b^	0.08 ± 0.00 ^B,b^	0.09 ± 0.00 ^AB,ab^	n.d.	0.11 ± 0.00 ^AB,a^	**
	SIGN.	**	**	**	N.S.	*	
pH	1	6.36 ± 0.02 ^B,a^	6.26 ± 0.01 ^C,a^	6.26 ± 0.00 ^C,a^	5.84 ± 0.01 ^A,b^	5.97 ± 0.13 ^B,b^	**
	3	6.48 ± 0.08 ^B,b^	6.62 ± 0.05 ^AB,a^	6.51 ± 0.06 ^B,ab^	5.70 ± 0.01 ^B,d^	6.17 ± 0.00 ^B,c^	**
	7	6.67 ± 0.13 ^AB,a^	6.41 ± 0.25 ^BC,a^	6.59 ± 0.01 ^B,a^	5.68 ± 0.04 ^B,b^	6.50 ± 0.11 ^A,a^	**
	10	6.91 ± 0.06 ^A,a^	6.87 ± 0.04 ^A,a^	6.84 ± 0.01 ^A,a^	n.d.	6.46 ± 0.13 ^A,b^	**
	14	6.66 ± 0.28 ^AB,a^	6.91 ± 0.11 ^A,a^	6.75 ± 0.10 ^A,a^	n.d.	6.18 ± 0.03 ^B,b^	**
	SIGN.	**	**	**	**	**	
Total soluble solids	1	2.95 ± 0.07 ^b^	1.90 ± 0.14 ^B,c^	1.75 ± 0.07 ^B,c^	3.05 ± 0.07 ^B,ab^	3.25 ± 0.07 ^a^	**
	3	3.05 ± 0.35	2.75 ± 0.64 ^AB^	3.00 ± 0.14 ^A^	3.65 ± 0.64 ^AB^	4.30 ± 0.85	n.s.
	7	3.10 ± 0.42 ^ab^	2.30 ± 0.42 ^AB,b^	2.50 ± 0.42 ^AB,b^	3.90 ± 0.14 ^A,a^	3.70 ± 0.57 ^a^	**
	10	2.80 ± 0.00 ^ab^	2.05 ± 0.07 ^AB,b^	3.35 ± 0.78 ^A,a^	n.d.	3.50 ± 0.57 ^a^	**
	14	2.60 ± 0.28 ^b^	2.90 ± 0.14 ^A,b^	2.75 ± 0.21 ^A,b^	n.d.	3.65s ± 0.35 ^a^	**
	SIGN.	N.S.	*	**	**	N.S.	
Dry matter	1	4.07 ± 0.44 ^b^	4.26 ± 0.42 ^AB,b^	4.06 ± 0.69 ^b^	5.54 ± 0.03 ^a^	6.06 ± 0.37 ^a^	**
	3	3.77 ± 1.12 ^b^	4.08 ± 0.42 ^AB,ab^	4.18 ± 0.28 ^ab^	6.02 ± 1.19 ^a^	5.26 ± 0.19 ^ab^	*
	7	4.72 ± 0.84 ^ab^	4.84 ± 0.17 ^A,ab^	3.67 ± 1.58 ^b^	6.41 ± 0.67 ^a^	5.14 ± 0.32 ^ab^	*
	10	4.16 ± 0.29 ^b^	4.58 ± 0.28 ^AB,ab^	5.04 ± 0.12 ^ab^	n.d.	5.31 ± 0.79 ^a^	**
	14	4.17 ± 0.59	3.96 ± 0.01 ^B^	4.84 ± 0.36	n.d.	5.34 ± 1.09	n.s.
	SIGN.	N.S.	*	N.S.	N.S.	N.S.	

Data are presented as the means ± SD (n = 3). Means within a row or column with different letters are significantly different by Tukey’s post hoc test. Lowercase letters indicate differences among treatments for a set treatment time. Uppercase letters indicate differences among treatment times for a set treatment. Abbreviations: Sign. and SIGN., significance; n.s. and N.S, not significant; n.d., not detected; ** significance at *p* < 0.01; * significance at *p* < 0.05. UCR, uncoated minimally processed radishes; DRa, minimally processed radishes dipped in 0.3% citric acid solution, *w*/*v*; DRb, minimally processed radishes dipped in 1% LP_E_ solution, *v*/*v*; CRc, minimally processed radishes coated with an alginate-based coating (2%, *w*/*v*); CRd, minimally processed radishes coated with an alginate-based coating (2%, *w*/*v*) with LP_E_ (1%, *v*/*v*).

**Table 3:** Hue angle (h°) values of uncoated and coated minimally processed radishes throughout 14 days of storage at 3 °C.

Inner							
	Days	1	3	7	10	14	SIGN.
Sample							
UCR		72.69 ± 0.30 ^C,b^	64.39 ± 0.22 ^E,b^	71.58 ± 0.09 ^D,c^	75.30 ± 0.27 ^B,d^	78.52 ± 0.14 ^A,c^	**
DRa		76.57 ± 0.28 ^C,a^	59.36 ± 0.32 ^D,c^	76.71 ± 0.18 ^C,b^	84.42 ± 0.14 ^A,a^	81.66 ± 0.41 ^B,b^	**
DRb		77.26 ± 0.32 ^C,a^	56.51 ± 0.29 ^E,d^	80.31 ± 0.21 ^B,a^	81.71 ± 0.40 ^A,b^	74.47 ± 0.15 ^D,d^	**
CRc		30.68 ± 0.23 ^A,d^	26.60 ± 0.16 ^B,e^	19.36 ± 0.36 ^C,d^	n.d.	n.d.	**
CRd		40.59 ± 0.34 ^E,c^	68.56 ± 0.23 ^D,a^	80.45 ± 0.23 ^B,a^	76.28 ± 0.15 ^C,c^	85.60 ± 0.35 ^A,a^	**
Sign.		**	**	**	**	**	
**Outer**							
	**Days**	**1**	**3**	**7**	**10**	14	**SIGN.**
**Sample**							
UCR		11.16 ± 0.22 ^A,b^	10.61 ± 0.23 ^A,c^	7.57 ± 0.10 ^B,c^	−2.10 ± 0.14 ^D,d^	3.61 ± 0.12 ^C,c^	**
DRa		13.55 ± 0.15 ^A,a^	12.63 ± 0.14 ^B,b^	9.46 ± 0.10 ^C,b^	2.58 ± 0.04 ^D,c^	−0.28 ± 0.09 ^E,d^	**
DRb		13.67 ± 0.12 ^B,a^	14.62 ± 0.31 ^A,a^	9.46 ± 0.21 ^C,b^	10.23 ± 0.13 ^C,a^	6.71 ± 0.24 ^D,b^	**
CRc		11.21 ± 0.26 ^C,b^	15.28 ± 0.19 ^B,a^	23.28 ± 0.16 ^A,a^	n.d.	n.d.	**
CRd		10.18 ± 0.23 ^B,c^	8.58 ± 0.22 ^C,d^	6.22 ± 0.23 ^E,d^	7.47 ± 0.28 ^D,b^	12.59 ± 0.23 ^A,a^	**
Sign.		**	**	**	**	**	

Data are presented as the means ± SD (n = 10). Means within a row or column with different letters are significantly different by Tukey’s post hoc test. Lowercase letters indicate differences among treatments for a set treatment time. Uppercase letters indicate differences among treatment times for a set treatment. Abbreviations: n.d.; Sign.; SIGN.; **; *; UCR; DRa; DRb; CRc; CRd (see Table 2).

**Table 4:** Total polyphenol content (TPC), total anthocyanin content (TAC) and antioxidant activity values (DPPH and ABTS assays) of uncoated and coated minimally processed radishes throughout 14 days of storage at 3 °C.

	Days	UCR	DRa	DRb	CRc	CRd	SIGN.
TPC (mg GAE kg^−1^)	1	399 ± 79	382 ± 25	433 ± 46	443 ± 12	467 ± 65	N.S.
	3	397 ± 59	402 ± 28	442 ± 29	380 ± 47	440 ± 10	N.S.
	7	428 ± 32	460 ± 53	429 ± 24	493 ± 15	493 ± 16	N.S.
	10	374 ± 21	373 ± 7	400 ± 25	n.d.	362 ± 47	N.S.
	14	389 ± 26	334 ± 19	424 ± 72	n.d.	393 ± 7	N.S.
	Sign.	n.s.	n.s.	n.s.	n.s.	n.s.	
TAC (mg C-3-Glu kg^−1^)	1	195 ± 37	205 ± 16	236 ± 49	200 ± 9 ^a^	246 ± 23 ^a^	N.S.
	3	202 ± 45	211 ± 41	235 ± 18	137 ± 8 ^b^	225 ± 17 ^ab^	N.S.
	7	241 ± 28	265 ± 35	225 ± 8	197 ± 0 ^a^	264 ± 7 ^a^	N.S.
	10	202 ± 14 ^A^	206 ± 7 ^A^	199 ± 7 ^A^	n.d.	160 ± 4 ^B,c^	*
	14	205 ± 0 ^A^	162 ± 1 ^B^	194 ± 4 ^A^	n.d.	189 ± 9 ^A,bc^	**
	Sign.	n.s.	n.s.	n.s.	**	**	
DPPH (inactivation %)	1	34.26 ± 5.31	34.28 ± 1.17 ^ab^	37.18 ± 5.63	22.87 ± 4.08	33.34 ± 5.17	N.S.
	3	33.50 ± 1.79 ^A^	34.37 ± 0.43 ^A,ab^	34.92 ± 0.86 ^A^	21.87 ± 0.16 ^B^	34.52 ± 1.94 ^A^	**
	7	38.04 ± 4.60 ^AB^	38.08 ± 2.59 ^A,a^	38.62 ± 4.92 ^A^	18.79 ± 5.20 ^B^	30.31 ± 6.02 ^AB^	*
	10	36.05 ± 7.37	34.49 ± 0.88 ^ab^	35.00 ± 3.18	n.d.	23.62 ± 2.35	N.S.
	14	29.53 ± 4.69	27.28 ± 2.98 ^b^	33.09 ± 0.11	n.d.	27.98 ± 2.37	N.S.
	Sign.	n.s.	*	n.s.	n.s.	n.s.	
ABTS (inactivation %)	1	32.47 ± 5.64	32.52 ± 0.86	38.45 ± 2.32	29.84 ± 4.33	36.71 ± 2.04	N.S.
	3	29.57 ± 0.70	34.59 ± 1.44	34.07 ± 6.43	21.66 ± 2.09	33.26 ± 6.35	N.S.
	7	46.55 ± 5.84	39.64 ± 2.01	35.65 ± 6.21	30.31 ± 6.98	40.23 ± 6.70	N.S.
	10	31.88 ± 4.07 ^AB^	32.68 ± 1.58 ^AB^	35.85 ± 0.89 ^A^	n.d.	23.72 ± 1.72 ^B^	*
	14	36.48 ± 8.84	32.96 ± 4.34	41.29 ± 2.52	n.d.	31.42 ± 0.74	N.S.
	Sign.	n.s.	n.s.	n.s.	n.s.	n.s.	

Data are presented as the means ± SD (n = 3). Means within a row or column with different letters are significantly different by Tukey’s post hoc test. Uppercase letters indicate differences among treatments for a set treatment time. Lowercase letters indicate differences among treatment times for a set treatment. Abbreviations: Sign. and SIGN.; n.s. and N.S.; n.d.; **; *; UCR; DRa; DRb; CRc; CRd (see Table 2).

**Table 5:** Main phenolic compounds identified and quantified in minimally processed radish samples (mg kg^−1^) by the UHPLC system.

		Quercetin	Ferulic Acid	Luteolin	Quercetin 3,4 Glucoside
1^st^ day	UCR	13.20 ± 0.20 ^c^	2.04 ± 0.01 ^c^	37.88 ± 0.14 ^d^	17.62 ± 0.19 ^d^
	DRa	13.13 ± 0.14 ^c^	2.03 ± 0.09 ^c^	39.43 ± 0.02 ^c^	17.41 ± 0.26 ^d^
	DRb	21.74 ± 0.03 ^a^	4.13 ± 0.11 ^b^	43.92 ± 0.11 ^b^	27.49 ± 0.08 ^a^
	CRc	10.03 ± 0.17 ^d^	2.33 ± 0.21 ^c^	39.25 ± 0.28 ^c^	21.08 ± 0.10 ^c^
	CRd	16.15 ± 0.10 ^b^	5.05 ± 0.03 ^a^	44.46 ± 0.01 ^a^	25.83 ± 0.02 ^b^
	Sign.	**	**	**	**
14^th^ day	UCR	19.13 ± 0.12 ^a^	2.16 ± 0.01 ^b^	42.10 ± 0.34 ^a^	23.95 ± 0.09 ^b^
	DRa	13.54 ± 0.03 ^b^	1.71 ± 0.22 ^c^	40.35 ± 0.06 ^b^	22.47 ± 0.10 ^c^
	DRb	8.42 ± 0.01 ^c^	3.94 ± 0.03 ^a^	40.70 ± 0.23 ^b^	24.71 ± 0.31 ^a^
	CRc	6.22 ± 0.17 ^e^	1.63 ± 0.18 ^c^	39.01 ± 0.05 ^c^	20.45 ± 0.03 ^d^
	CRd	7.48 ± 0.12 ^d^	2.54 ± 0.21 ^b^	41.57 ± 0.24 ^a^	22.41 ± 0.11 ^c^
	Sign.	**	**	**	**

Data are presented as the means ± SD (n = 3). Means within a column with different letters are significantly different by Tukey’s post hoc test. Abbreviations: Sign.; n.s.; **; *; UCR; DRa; DRb; CRc; CRd (see Table 2).

## Data Availability

Data are contained within the article.
